# Cluster of Sylvatic Epidemic Typhus Cases Associated with Flying Squirrels, 2004–2006

**DOI:** 10.3201/eid1507.081305

**Published:** 2009-07

**Authors:** Alice S. Chapman, David L. Swerdlow, Virginia M. Dato, Alicia D. Anderson, Claire E. Moodie, Chandra Marriott, Brian Amman, Morgan Hennessey, Perry Fox, Douglas B. Green, Eric Pegg, William L. Nicholson, Marina E. Eremeeva, Gregory A. Dasch

**Affiliations:** Centers for Disease Control and Prevention, Atlanta, Georgia, USA (A.S. Chapman, D.L. Swerdlow, A.D. Anderson, C.E. Moodie, B. Amman, M. Hennessey, D.B. Green, E. Pegg, W.L. Nicholson, M.E. Eremeeva, G.A. Dasch); Pennsylvania Department of Health, Pittsburgh, Pennsylvania, USA (V.M. Dato, C. Marriott, P. Fox); 1 Current affiliation: US Air Force, Scott Air Force Base, Illinois, USA.

**Keywords:** Sylvatic typhus, epidemic typhus, flying squirrel, Rickettsia prowazekii, rickettsia, zoonoses, CME, podcast, Pennsylvania, research

## Abstract

In February 2006, a diagnosis of sylvatic epidemic typhus in a counselor at a wilderness camp in Pennsylvania prompted a retrospective investigation. From January 2004 through January 2006, 3 more cases were identified. All had been counselors at the camp and had experienced febrile illness with myalgia, chills, and sweats; 2 had been hospitalized. All patients had slept in the same cabin and reported having seen and heard flying squirrels inside the wall adjacent to their bed. Serum from each patient had evidence of infection with *Rickettsia prowazekii*. Analysis of blood and tissue from 14 southern flying squirrels trapped in the woodlands around the cabin indicated that 71% were infected with *R. prowazekii*. Education and control measures to exclude flying squirrels from housing are essential to reduce the likelihood of sylvatic epidemic typhus.

## Abstract 2

Infected persons had slept in an infested cabin.

Sylvatic epidemic typhus, hereafter referred to as sylvatic typhus, is a rare but potentially lethal zoonotic exanthematous disease caused by *Rickettsia prowazekii*. It is associated with a cycle of infection involving flying squirrels and their ectoparasites and secondary transmission to humans. Illness in humans is characterized by fever, myalgia, severe headache, and rash. Historically, classic louse-borne epidemic typhus, caused by the same organism, has caused large epidemics where conditions were favorable for person-to-person spread of body lice.

Surveillance for epidemic typhus was discontinued in the United States in the 1950s because the illness had not been reported in decades and prevalence of body lice infestation in this country has been low (*1*). However, in 1975, the southern flying squirrel, *Glaucomys volans*, was found to be naturally infected with *R. prowazekii* (*2*). Later studies demonstrated serologic evidence of human *R. prowazekii* infection associated with flying squirrels in the eastern United States (*1*,*3*,*4*). Fleas and lice carried by the squirrels become naturally infected (*1*,*5*,*6*) and may be responsible for transmission (*7*); however, the exact mechanism of transmission remains unknown. From 1976 through 2002, a total of 41 cases of human *R. prowazekii* infection were documented in persons who had no reported contact with body lice or persons infested with lice (*3*,*4*,*7*–*10*). Almost all cases occurred in the eastern United States. Approximately one third of the patients had confirmed contact with flying squirrels or their nests before disease onset (*4*,*7*); the remaining patients had no identified association with flying squirrels.

On February 23, 2006, serologic testing performed at the Centers for Disease Control and Prevention (CDC) identified a case of sylvatic typhus in a 31-year-old counselor at a therapeutic wilderness camp for troubled youth in Pennsylvania (case-patient 1). A review of the Pennsylvania electronic disease surveillance system found that in December 2004 another counselor from the same camp (case-patient 2) had become ill with fever, chills, and sweats and that serologic testing at a local laboratory had been suggestive of sylvatic typhus. During a 25-year period (1976−2001), only 2 other cases of sylvatic typhus associated with flying squirrels had been reported in Pennsylvania (*7*). Laboratory and epidemiologic investigations of humans and animals associated with the camp were conducted to identify additional sylvatic typhus cases at the camp, risk factors for infection, and prevalence of *R. prowazekii* in the natural host. A total of 4 cases of sylvatic typhus were found to have occurred during the 24-month period of January 2004–January 2006. All were linked epidemiologically to a specific cabin and bed at the Pennsylvania camp. The investigation also found laboratory evidence of *R. prowazekii* infection in the population of flying squirrels at the camp.

## Methods

### Epidemiologic Investigation

We defined a suspected case of sylvatic typhus as fever (>38°C) plus >1 of the following: headache, myalgia, confusion, rash, or photophobia, with illness onset prior to March 1, 2006, in a staff member or student at the camp. A confirmed case of sylvatic typhus was defined as a case that met the definition of a suspected case plus had immunoglobulin (Ig) M and/or IgG reactive with whole-cell *R. prowazekii* antigen at a dilution >1:64, according to immunofluorescence antibody (IFA) assay performed at CDC (*11*). Persons who met the definition for having a suspected case but who had negative serologic results for *R. prowazekii* were not considered to have a confirmed case of sylvatic typhus. Interviews were conducted to ascertain exposure history, and medical records were reviewed. In March 2006, to identify other possible cases of sylvatic typhus and to assess risk factors for infection, a standardized questionnaire was administered to the camp staff and serum samples were obtained. The questionnaire gathered information about job classification, length of employment, cabin sleeping history, and contact with flying squirrels or their nests. Medical records of enrolled students (typically at the camp for 9–18 months) were assessed to identify illness suggestive of sylvatic typhus occurring during their residence. Records of students discharged from the program before February 2006 were not available for review. Data analyses were performed by using Epi Info 2000 (*12*). Odds ratios, confidence intervals, and p values were calculated by using SAS version 9.1.3. (SAS Institute, Inc., Cary, NC, USA) (*13*).

### Environmental Investigation

During the winter (October–March), staff and students had lived in 1 of 6 cabins (cabins A–F); during the rest of the year, they had slept in tents (Figure). All 6 cabins were inspected for evidence of animal infestation, including flying squirrels, and for openings where flying squirrels could enter the cabins. In March 2006, baited live trapping using Sherman box traps (H.B. Sherman Traps, Tallahassee, FL, USA) was conducted on 5 consecutive nights throughout the camp. Trapping sites included a tent site occupied only during summer; sites of cabins A, B, and F; and 2 nearby sites not directly associated with the cabins.

**Figure:**
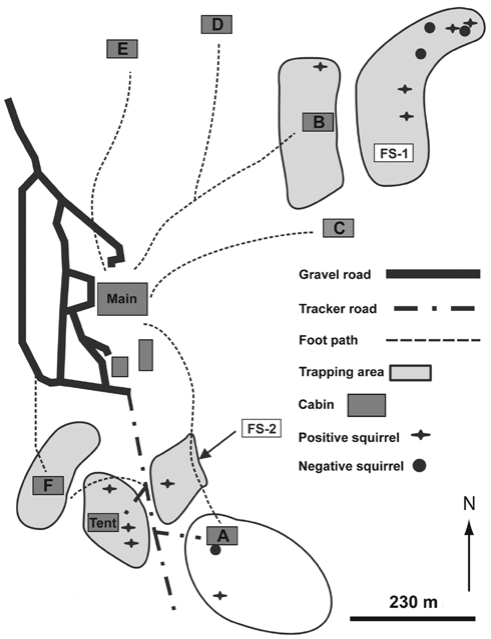
Wilderness camp, Pennsylvania, USA, showing areas where flying squirrels were trapped over a 5-day period during March 2006 for *Rickettsia prowazekii* testing. Cabins and tent sites are designated by letters A, B, C, D, E, F, and Tent. Field sites are designated FS-1 and FS-2.

Blood was obtained from trapped flying squirrels for detection of IgG (heavy plus light chain) polyvalent antibodies against *R. prowazekii* by IFA. Serologic testing was performed at CDC with an anti−flying squirrel fluorescein-isothiocyanate conjugate prepared in-house. Samples were first screened at 1:32 dilution; any reactive samples were titered to end point. Necropsies were performed and tissues were collected, frozen on dry ice, and kept at –70°C before further testing. DNA was prepared from whole blood and heart tissues by using QIAamp DNA Mini Kit (QIAGEN, Valencia, CA, USA) according to manufacturer’s instructions. PCRs to amplify a fragment of 17-kDa protein antigen were carried out in a total volume of 25 μL by using Taq Master Mix (QIAGEN) in a Gradient Mastercycler (Eppendorf, Westbury, NY, USA). Details of the PCR setup and primer sequences are described elsewhere (*14*). Identity of the PCR amplicon was confirmed by direct sequencing performed using an ABI PRISM 3.0 BigDye Terminator Cycle Sequencing kit as recommended by manufacturer (Applied BioSystems, Foster City, CA, USA). Nucleotide sequences generated during this study were submitted to the NCBI GenBank.

## Results

Of 66 camp staff members on site during the March 2006 investigation, 45 (68%) completed a questionnaire; 33 of the 45 (73%) provided a serum sample, including 17 staff members who met the case definition for suspected sylvatic typhus. Five additional staff members reported having had febrile illness since employment at the camp but declined testing; 2 of them met the definition for suspected sylvatic typhus. Serologic testing confirmed 4 (12%) cases of sylvatic typhus among the 33 tested staff members, including case-patients 1 and 2, who were identified at the start of the investigation (Table 1). The remaining 13 staff members who met the definition of having a suspected case but who had no serologic evidence of *R. prowazekii* infection were classified as non–case-patients. Two persons who had suspected cases but did not undergo serologic testing could not be further classified and were grouped with other survey-only respondents for the purpose of analysis.

**Table 1:** Characteristics of patients with flying squirrel–associated sylvatic typhus, Pennsylvania, USA, 2004–2006*

Patient no. (age, y)*	Date of illness onset	Exposure‡									Hospitalized
		Touched flying squirrel	Slept in bunk B		Serologic titer (date)			Clinical sign§			
					IgG	IgM		Fever, °C	Headache	Photophobia	
1 (31)	2006 Jan	No	A		512 (2006 Feb)	4,096 (2006 Feb)		39.6	Yes	No	Yes
2 (39)	2004 Dec	Yes	F		1,024 (2006 Mar)	512 (2006 Mar)		40.3	Yes	Yes	Yes
3 (26)	2005 Jan	No	F		128 (2006 Mar)	ND		39.4	No	No	No
4 (26)	2005 Dec	No	S		256 (2006 Mar)	ND		39.8	Yes	Yes	No

*Ig, immunoglobulin titer against *Rickettsia prowazekii;* ND, not done. †All patients were male. ‡All patients lived in cabin A. A, always; F, frequently; S, sometimes. §All patients had chills and muscle pain but not rash.

### Case Reports

#### Case 1

On January 7, 2006, a 31-year-old man from western Pennsylvania became ill with high fever (39.6°C), chills, sweats, headache, and joint and muscle aches. The next day, he was admitted to the hospital. During his hospitalization, he continued to have fever and muscle aches, and laboratory test results were positive for mononucleosis and influenza A. He was discharged on January 17 but continued to have headache and fever. After hearing of a case of sylvatic typhus at the camp where he worked the previous year, he requested testing from his hospital clinician. On January 24, he was reevaluated and serum was obtained for sylvatic typhus testing. He was given oral doxycycline, 100 mg twice a day for 10 days; his signs and symptoms resolved. The man had lived in cabin A and stated that although he had never handled a flying squirrel, he had heard them frequently in the wall next to his bunk (bunk B) (Figure).

#### Case 2

On December 27, 2004, a 39-year-old man from southwestern Pennsylvania had fever (40.3°C), chills, sweats, headache, and muscle and joint pain. He later became confused and was admitted to the hospital on January 4, 2005. At the time of admission, he was febrile and dehydrated. He was treated with azithromycin and ciprofloxacin but did not improve. When he told clinicians that he worked at a wilderness camp and had been bitten by fleas, he was administered a regimen of intravenous doxycycline for 3 days. His fever subsequently resolved; he was discharged from the hospital in early January and given oral doxycycline, 100 mg twice a day for 7 days. He visited his primary care provider in February 2005. IgG and IgM tests performed at a commercial laboratory on serum collected on February 1, 2005, showed qualitatively positive results for typhus group–specific antibodies. The man had lived in cabin A at the time of his illness. He reported having seen a flying squirrel 1 time in the main living area of the cabin and seeing squirrels in the walls next to his bunk. He also reported feeding the squirrels through wide gaps in the wall boards next to his bunk (bunk B) and being bitten by insects he recognized as fleas in his bunk at least 1 time the previous year.

#### Case 3

In January 2004, a 26-year-old man from Pennsylvania had high fever (39.4°C), chills, sweats, muscle aches, nausea, and vomiting. The man did not seek medical attention and recovered within a week. He worked as a counselor at the camp in Pennsylvania and, at the time of his illness, was living in cabin A. He reported that he had seen animals inside gaps in the wall next to his bunk (bunk B) and had seen a flying squirrel in the main living area of the cabin on 1 occasion.

#### Case 4

In late December 2005, a 26-year-old man from Pennsylvania had high fever (39.8°C), severe headache, muscle and joint pain, chills, sweats, and photophobia. He did not seek medical attention and recovered after 2 weeks. He worked as a counselor at the camp and, at the time of his illness, lived in cabin A. He reported having seen and heard animals inside the wall next to his bunk (bunk B) many times and attempting to seal gaps in the wall with foam caulking spray. Although he had never touched a flying squirrel, he had seen one traversing a roof truss inside cabin A, had handled nesting material, and had been bitten by insects he recognized as fleas while living in the cabin before his illness.

The median age of all 45 questionnaire respondents was 34 years (range 21–69 years); 97% were white, and 84% were male. The median length of employment at the camp was 2 years (range 1 month–31 years). Selected characteristics reported are summarized in Table 2. Of 45 (69%) respondents, 31 reported seeing gaps between interior wall boards in all cabins. Gaps were most frequently observed in cabin A (24/31; 77%), followed by cabins D (13/31; 42%), F (12/31; 38%); E (10/31; 32%); and B and C (each 9/31; 29%) (Figure). Seeing flying squirrels in a cabin was reported by 15 (33%) respondents; however, 7 could not recall which cabin. Among the 8 who recalled the location, 5 (63%) reported seeing them in cabin A and 1 each recalled seeing them in cabins B, C, and D.

**Table 2:** Selected characteristics of surveyed staff members at wilderness camp in Pennsylvania, March 2006*

Characteristic	Case-patients (n = 4)	Non–case-patients (n = 29)	Case-patients surveyed† (n = 12)
Median age, y‡	28.5 (range 26–40)	38.7 (range 21–69)	35 (range 23–63)
Gender			
Male‡	4 (100)	23 (79)	11 (92)
Female	0	6 (21)	1 (8)
Job classification			
Counselor‡	4 (100)	14 (48)	7 (58)
Maintenance	0	3 (10)	1 (8.3)
Administrative	0	5 (17)	1 (8.3)
Support staff	0	5 (17)	1 (8.3)
Night security	0	1 (3.4)	2 (17)
No answer	0	1 (3.4)	0
Employment, y‡	2.5 (range 1.8–3.9)	2.7 (range 0.1–30.8)	1.1 (range 0.2–26.3)
Ever slept in cabins			
Yes‡	4 (100)	19 (66)	8 (66.6)
No	0	10 (34)	3 (25)
Don’t know	0	0	1 (8.3)
Contact with (touching) flying squirrel			
Yes‡	1 (25)	7 (24)	1 (8)
No	3 (75)	20 (69)	11 (92)
Don’t know	0	1 (3.4)	0
No answer	0	1 (3.4)	0
Contact with (touching) flying squirrel nests§			
Yes	2 (50)	3 (10)	0
No	1 (25)	17 (58.6)	10 (83)
Don’t know	1 (25)	8 (28)	2 (17)
No answer	0	1 (3.4)	0
Ever slept in cabin A			
Yes‡	4 (100)	13 (45)	4 (33)
No	0	16 (55)	8 (67)
Ever slept in bunk B in cabin A			
Yes¶	4 (100)	9 (69)	2 (50)
No	0	3 (23)	1 (25)
No answer	0	1 (7.6)	1 (25)
Cumulative months in cabin A#	17.00 (range 8–43)	2.00 (range 0–13)	0.25 (range 0–30)

*Data given as frequency (%) except as indicated. †Responses of staff members who declined serologic testing. Remaining respondents classified as case-patients or non–case-patients, according to results of serologic testing for *Rickettsia* *prowazekii* and patients’ medical histories. ‡These characteristics did not demonstrate statistical difference between case-patients and non–case-patients. §Odds ratio 11.3 (95% confidence interval 0.51–474) for association between contact with flying squirrel nests and sylvatic typhus. ¶Association with sleeping in bunk B of cabin A significant at p = 0.02. #Significant at p = 0.04.

The 4 confirmed case-patients had lived in cabin A for a significantly longer cumulative period (median 17 months) than non–case-patients (median 2 months; p = 0.04). Bunk B had been slept in by all 4 case-patients but by only 9 of 28 non–case-patients (1 non–case-patient unknown; p = 0.02, by Fischer exact test). From January 2004 through January 2006, 8 (18%) staff members lived primarily in cabin A, and other staff members occasionally slept in cabin A.

Although the students living in cabin A were not tested for typhus, a review of their medical records indicated that none had sought medical care for a febrile illness at any time during their enrollment. Of 43 students whose records were reviewed, 7 (16%) had experienced febrile illness compatible with sylvatic typhus since their arrival at the camp. These students resided in cabins D, E, and F. All 6 (86%) who were tested for evidence of *R. prowazekii* infection had negative results.

### Environmental Investigation

At the time of the March 2006 investigation, counselors and students were living in 5 of 6 cabins located on a 1,500 acre tract of land in southwestern Pennsylvania (Figure). The cabins were widely separated across the tract. Each site was occupied by ≈2 counselors and 10 students, who worked and attended school at the site and interacted with each other during events and meals. The land is mountainous and covered with a forest of mixed northern hardwood, predominantly oak, maple, black cherry, and yellow poplar. The cabins consisted of rough-cut wood planking on wooden stud construction. The roof was covered with asphalt shingles. Inside, 8–10 wood-framed bunk beds (for students) were primarily located against the cabin walls, surrounding a centrally placed wood-burning stove. A small alcove in each cabin, containing 2 bunk beds each set against a wall, was designated for counselors’ use. Bathing facilities were located in a separate building. Exterior walls were covered in horizontal, planed lumber. The inside walls were constructed of vertical planed lumber. Fiberglass insulation batting was installed in the hollow spaces in the wall.

When examined, the cabins at sites A, C, D, and F had evidence of deer mice and flying squirrel infestation, including nesting material protruding from the walls and ceiling and damaged or chewed insulation in walls adjacent to counselor bunk A (cabin C), counselor bunk B (cabin A), and student bunks (cabin D). A large opening providing access to the exterior of the cabin was present on the wall above the counselor bunk B in cabin A. In April 2006, camp staff removed the interior wooden planks from the wall adjacent to bunk B in cabin A and confirmed the presence of flying squirrel nesting materials and extensive damage to insulation inside the wall.

### Evidence of *R. prowazekii* Infection in Trapped Flying Squirrels

Trapping efforts obtained 14 southern flying squirrels from several outdoor sites throughout the camp, including the site where cabin A was located and a field site adjacent to a state park (Figure; Table 3). Of the 14 squirrels, 8 (57%) had antibodies to *R. prowazekii*, 6 with end-point titers from 32 to 1,024 (Table 4). These 8 squirrels had been captured at a tent site in the camp woodlands (n = 3), field sites not associated with cabins (n = 4), and a path leading to cabin A (n = 1). *R. prowazekii* DNA was detected in specimens (2 hearts and 3 whole blood samples) from 5 (36%) squirrels. Of the 14 squirrels, 3 showed both serologic and molecular evidence of infection and 12 hosted fleas or lice (data not shown).

**Table 3:** Total arboreal trapping effort and captures of flying squirrels, wilderness camp, Pennsylvania, March 2006*

Site	Traps	Trap-nights†	Captures	%‡
Cabin A	36	126	2	1.5
Cabin B	30	120	1	0.8
Cabin F	23	100	0	0
Tent	32	94	3	3.2
FS-1	30	90	7	7.8
FS-2	15	60	1	1.7
Total	166	500	14	2.8

*FS, field site. †Trap-night = 1 trap/1 night of effort. 5 traps for 1 night = 5 trap-nights. ‡No. captures divided by no. trap-nights.

**Table 4:** Serologic and PCR testing results of trapped flying squirrels, wilderness camp, Pennsylvania, March 2006*

Squirrel (ID)	Site trapped	IgG titer† to *Rickettsia prowazekii*	PCR result (specimen)
1 (P19)	Tent	512	– (blood)
2 (A59)	FS-1	32	– (blood)
3 (A60)	FS-1	<16	– (heart, blood)
4 (P1)	Tent	64	+ (heart)
5 (P28)	Tent	32	– (blood)
6 (A41)	FS-1	128	– (blood)
7 (A53)	FS-1	<16	– (blood)
8 (C4)	Cabin A	<16	– (blood)
9 (A43)	Cabin B	<16	+ (heart)
10 (A26)	FS-1	1,024	+ (blood)
11 (A56)	FS-1	<16	– (blood)
12 (A60h)	FS-1	<16	+ (blood)
13 (H12)	FS-2	64	+ (blood)
14 (C52)	Cabin A	128	– (blood)

*Ig, immunoglobulin; ID, identification number; FS, field site. †Heavy plus light chain.

## Discussion

Our investigation found that transmission of sylvatic typhus to humans occurred during 3 consecutive winters at the camp. All 4 cases among counselors were epidemiologically linked to cabin A. All had slept in a specific bunk, bunk B, next to a wall that had evidence of flying squirrel infestation. Other counselors sleeping in the same cabin but without direct exposure to bunk B had no evidence of infection. The finding that 10 (71%) of 14 flying squirrels collected at multiple sites showed evidence of *R. prowazekii* infection indicates that the pathogen is well established among these squirrels.

Although inhalation and transdermal or mucous membrane exposure to infected louse feces are well-established routes of transmission during epidemics of louse-borne typhus, the mechanism by which *R. prowazekii* is transmitted from flying squirrels to humans is not well understood. The lack of detectable antibodies to *R. prowazekii* in household members of documented sylvatic typhus case-patients (*6*,*8*) has been used to support the hypothesis that risk for sylvatic typhus in the absence of direct exposure to flying squirrels and their nests is low (*9*) and thus may explain why human disease has been reported only sporadically. The cluster of cases described here suggests that where repeated and prolonged close exposure to flying squirrels and their nests occurs, potential for transmission can be high. Only 1 case-patient described here reported direct contact with flying squirrels; however, all case-patients slept for many nights next to a wall that was continuously inhabited by flying squirrels. Nesting material, dander, arthropod feces, or ectoparasites may have been introduced to the living area of the cabin through the many openings in this cabin wall and provided a source of infection for those sleeping in the bunk adjacent to the wall but not elsewhere in the cabin. Because cabins at this camp were occupied continuously during the fall and winter months by the same group of counselors, epidemiologic linking of several infections occurring at different times during consecutive winters was possible. No identified cases occurred during the summer when staff and students lived outdoors in tents, further suggesting that exposure was highly focal to cabin A.

During spring of 2006, cabin A was vacated by staff and students for remediation. External openings were closed by using wire hardware cloth to exclude squirrel entry. The inside wall boards of the cabin were removed, insulation was replaced, and new wall boards were installed. The entire cabin was professionally treated to eliminate ectoparasites. Staff and students were educated about sylvatic typhus and the need to avoid contact with flying squirrels and their nests. Since then, no additional cases of sylvatic typhus have been identified.

In contrast to classic epidemic typhus, which typically causes severe disease and mortality rates up to 4% despite antimicrobial drug therapy, fatal cases of typhus associated with flying squirrels have not been reported (*15*). Although flying squirrel isolates of *R. prowazekii* are reported to have biological, biochemical, and molecular properties similar to those of other typhus isolates (*16*–*18*), most cases of sylvatic typhus in the United States are apparently less severe than classic louse-borne epidemic typhus. All 4 patients with confirmed cases reported here had fever, headache, and malaise typical of sylvatic typhus; however, only 2 (case-patients 1 and 2) were hospitalized. None of the case-patients reported having had a rash with their illness. Rash has been reported for only half of patients with flying squirrel–associated typhus (*10*) and cannot be considered a reliable sign of the disease. Milder disease associated with sylvatic typhus may result from better nutritional and overall health status of most persons in the United States compared with those in populations affected by louse-borne epidemic typhus during war or other catastrophe. Because sylvatic typhus was not initially considered for any case-patients reported here, despite their occupational history, diagnosis and treatment were delayed. Given the nonspecific signs and symptoms associated with sylvatic typhus, the disease is likely underdiagnosed.

Clinicians should consider sylvatic typhus when evaluating a patient with compatible signs and symptoms and should inquire about potential exposures to flying squirrels. Clinicians who suspect sylvatic typhus on the basis of clinical presentation and history of potential exposure should empirically treat with doxycycline and not withhold treatment pending laboratory confirmation by serologic testing. Submission of blood or skin punch biopsy samples (when rash is present) for PCR analysis may provide options for earlier diagnosis, but these assays are not widely available. Serologic confirmation of infection is based on 4-fold IgG titer increases between acute and convalescent samples; both IgM and IgG can persist for years after infection with *R. prowazekii*.

Because southern flying squirrels are distributed throughout the eastern United States, other woodland settings frequented by humans during winter may present a similar risk for sylvatic typhus. Parks and campgrounds that maintain rental cabins should be aware of risks associated with flying squirrels and take steps to exclude these animals from structures occupied by humans by sealing openings at attic vents and around roof joists with heavy-gauge screen or similar products. In addition, use of repellents may keep arthropods from biting humans, and premise sprays may be useful for reducing arthropods in building structures. Squirrel removal without concomitant arthropod control is not recommended because the presence of potentially infected external parasites may increase the risk for disease transmission to humans.
